# Artificial iris performance for smart contact lens vision correction applications

**DOI:** 10.1038/s41598-020-71376-1

**Published:** 2020-09-04

**Authors:** Andrés Vásquez Quintero, Pablo Pérez-Merino, Herbert De Smet

**Affiliations:** 1 Centre for Microsystems Technology (CMST), Ghent University and Imec, Technologiepark 126, 9052 Ghent, Belgium; 2 Instituto de Investigación Sanitaria Fundación Jiménez Díaz, Avda. Reyes Católicos 2, 28040 Madrid, Spain

**Keywords:** Optoelectronic devices and components, Displays

## Abstract

This paper presents the simulated performance assessment of an artificial iris embedded on a scleral contact lens using real data from an aniridia patient. The artificial iris is based on guest–host liquid crystal cells (GH-LCD) in order to actively modify the transmittance of the lens and effective pupil size. Experimental validation of the GH-LCD spectrum and iris contrast (determined to be 1:2.1) enabled the development of optical models that include the effect of a small pupil on image quality and visual quality on an optical system with aniridia characteristics. Visual simulations at different light conditions (high/low photopic and mesopic) demonstrated the theoretical capacity of the customized artificial iris smart contact lens to expand the depth-of-focus and decrease the optical aberrations (in particular, the spherical aberration). The visual modelling suggests a maximum depth-of-focus value for a 2-mm pupil diameter for both eyes as follows: 3D (1,000 cd/m^2^), 2D (10 cd/m^2^) and 0.75D (1 cd/m^2^). This work demonstrates the beneficial optical effects of an active artificial iris, based on visual simulations in response to different light levels, and enables further experimental investigation on patients to validate the dynamic light attenuation and visual performance of smart contact lenses with GH-LCD.

## Introduction

A smart contact lens is a device with integrated electronics in direct contact with the eye, which provides sensing, actuation and wireless communication^1,2^ and offers both remarkable opportunities and challenges in a wide range of ocular applications: (1) active vision correction^3–8^, (2) biomedical sensing^9–11^ and (3) augmented reality^5,12^. These applications can be attained thanks to important breakthroughs in miniaturized stretchable systems and hybrid integration of a variety of components onto flexible platforms^13–19^. Liquid crystal cells (LC) are particularly attractive as an active electro-optical component^20,21^ for presbyopia correction (the age-related loss of the ability to dynamically focus near and far objects)^22,23^ or a solution for iris defects (such as aniridia, the partial or complete absence of iris)^8^.

The artificial iris solution is based on the so-called guest–host liquid crystal (GH-LCD)^24,25^. GH-LCD is composed by liquid crystal, chiral dopant and dichroic dye, and is capable of producing transmittance variations when an electric field is applied between its parallel electrodes, due to the combination of chirality and double absorption profiles of the mix^4^. The GH-LCD electrodes can be partitioned in independent rings that mimic the functionality of the natural iris when actuated in the correct sequence. Controlled transmittance changes of every ring make the GH-LCD technology an interesting approach to create an active artificial iris embedded within a contact lens; opening extraordinary possibilities in patients with aniridia by attenuating the light intensity through the ocular media and offering new options for presbyopia correction by expanding depth-of-focus with an automatic control of the pupil size.

The natural iris controls the pupil size in response to light, regulating the amount of light that reaches the retina and, thereby, visual perception. The natural human pupil changes by approximately 15-fold under varying levels of luminance, with dilation in low light conditions and constriction with bright light. The loss of this natural adaptive response may lead to photophobia, glare, halos, an increase of aberrations (monochromatic and chromatic) and, consequently, a decreased visual acuity^26^. In addition, during the accommodation reflex, the pupil constricts to increase the depth-of-focus of the eye. So, one simple way to increase the depth-of-focus and lower the retinal illuminance is with a fixed small central aperture in an ophthalmic solution (e.g., colored contact lenses or intraocular small-aperture inlays—recently introduced for presbyopia correction)^27^. However, these are passive solutions with potential limitations as de-centration, reduction in the visual field, decrease in the contrast sensitivity and artifacts in the retinal image quality (mainly due to the increased diffraction effect, distortions in the perception of relative movement or vignetting).

Unlike current passive solutions, an active artificial iris embedded in a contact lens is a non-invasive optical solution that could modulate automatically the ideal retinal illuminance by luminance changes in the visual environment, reduce the blur caused by high-order aberrations and increase the depth-of-focus as pupil size decreases. The ideal pupil function should be equal to one within the pupil area (modeled as a Gaussian-type function due to the directional sensitivity of the photoreceptors—apodization) and zero elsewhere (being blocked by the iris). However, to date GH-LCD contrast levels (i.e. between light passing through the rings when switched ON and OFF) are lower than 10:1^4^, this is due to the light leakage via the spacers and the limited spectral absorption of the dichroic dye during the ON state. While the GH-LCD smart contact lens can produce fast and automatic changes in the pupil size, some limitations associated with the relatively low contrast given by the GH-LCD could be expected for retinal illuminance in reading tasks but beneficial limiting photophobia in the case of aniridia.

The goal of this study is to measure, for the first time to our knowledge, in a laboratory setting the light transmission of the GH-LCD smart contact lens and the corresponding visual simulations at different light conditions (high photopic, low photopic and mesopic) considering three-dimensional real data from an aniridia patient. Particularly, we fabricate the GH-LCD active artificial iris and we demonstrate both experimentally and theoretically its feasibility as a potential optical solution for aniridia and theoretically its capacity to expand depth-of-focus.

## Results

### Qualitative optical quality and visible light transmittance (VLT)

**Figure 1:**
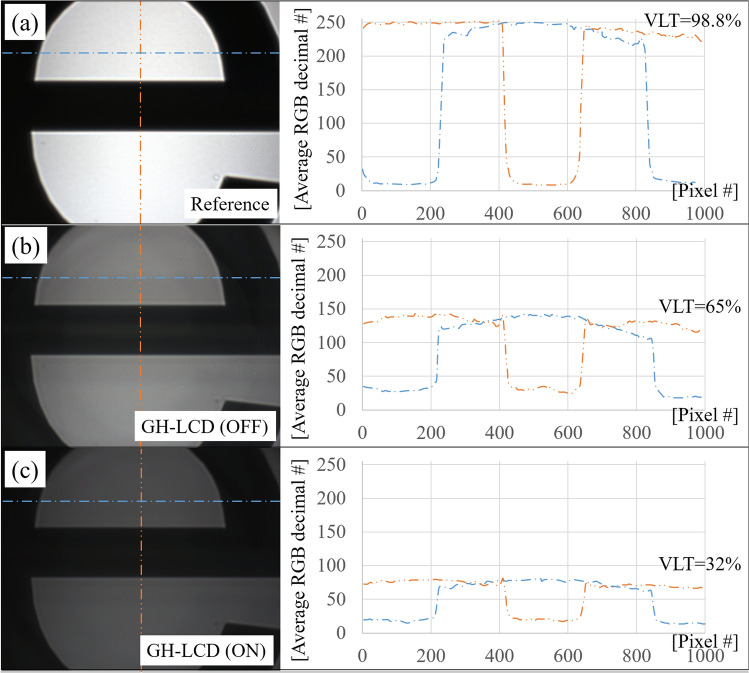
Captured images with the optical setup and CMOS camera for: (**a**) Reference image (without GH-LCD); (**b**) active GH-LCD during OFF state and (**c**) active GH-LCD during ON state. The right-hand side plots illustrate the average RGB decimal number for the pixels indicated with the dotted-lines, along a horizontal (single-dotted line) and vertical (double-dotted line) profiles, respectively.

Using a white light optical setup and CMOS camera, optical images of the stimulus were analyzed in order to assess their qualitative optical quality and VLT values during the operation of the active GH-LCD cells. Figure 1 shows the captured images (left) and the average decimal RGB (red, green and blue) color code with the VLT values (right), for the reference image, GH-LCD OFF state and GH-LCD ON state (along a vertical and horizontal profiles), respectively. The CMOS images clearly show a transmittance attenuation when looking through the active cells. The effect of light diffraction can also be observed by looking at the generated double border of the opaque features. This effect is caused by the intrinsic haze level of the substrate/conductor/GH-LCD and the diffraction of the static spacers, which in turn generate a blurry image. The inherent design of the micro-holes of the small aperture corneal inlays (KAMRA inlay has 8,400 pores of 5–11 μm diameter^28,29^, accounting for an area coverage of 8.6%) produce around 5% of diffraction through the permeable material^30^. However, this intrinsic drawback has recently turned into an advantage by exploiting the photon sieve concept^30,31^. A random spatial distribution (2,290–6,394) and a varying diameter (18.8–30.5 μm) of the micro-pores create a diffractive lens that brings near objects into focus^30,32^. Our spacer pattern distribution (900 spacers of 40 μm diameter; pitch 200 μm) covers only 3.5% of the GH-LCD area (28.3 mm^2^, for a diameter of 6 mm), which would in principle diffract less than 5%, compared to the KAMRA inlay. However, we did not explore the potential visual benefit of the spacer distribution on near focus nor its effect in the residual transmittance. A random distribution of spacers on the low transmittance region of the GH-LCD as opposed to a squared one is currently considered for future developments.

From Fig. 1, the VLT drops from 98.8 to 65% when the active cell (OFF state) is placed in the optical setup, reducing by half the light reaching the CMOS camera (corresponding to a contrast of 1:1.5). The VLT further drops to 32% when the active cell (ON state) is placed in the optical setup generating an absolute contrast of 1:3.1, with respect to the reference image. The relative contrast between the OFF and ON state is then calculated at 1:2 in this particular example. The relative contrast of the three cells measured was 1:2.1 with a standard deviation of 0.15.

### Spectral analysis

**Figure 2:**
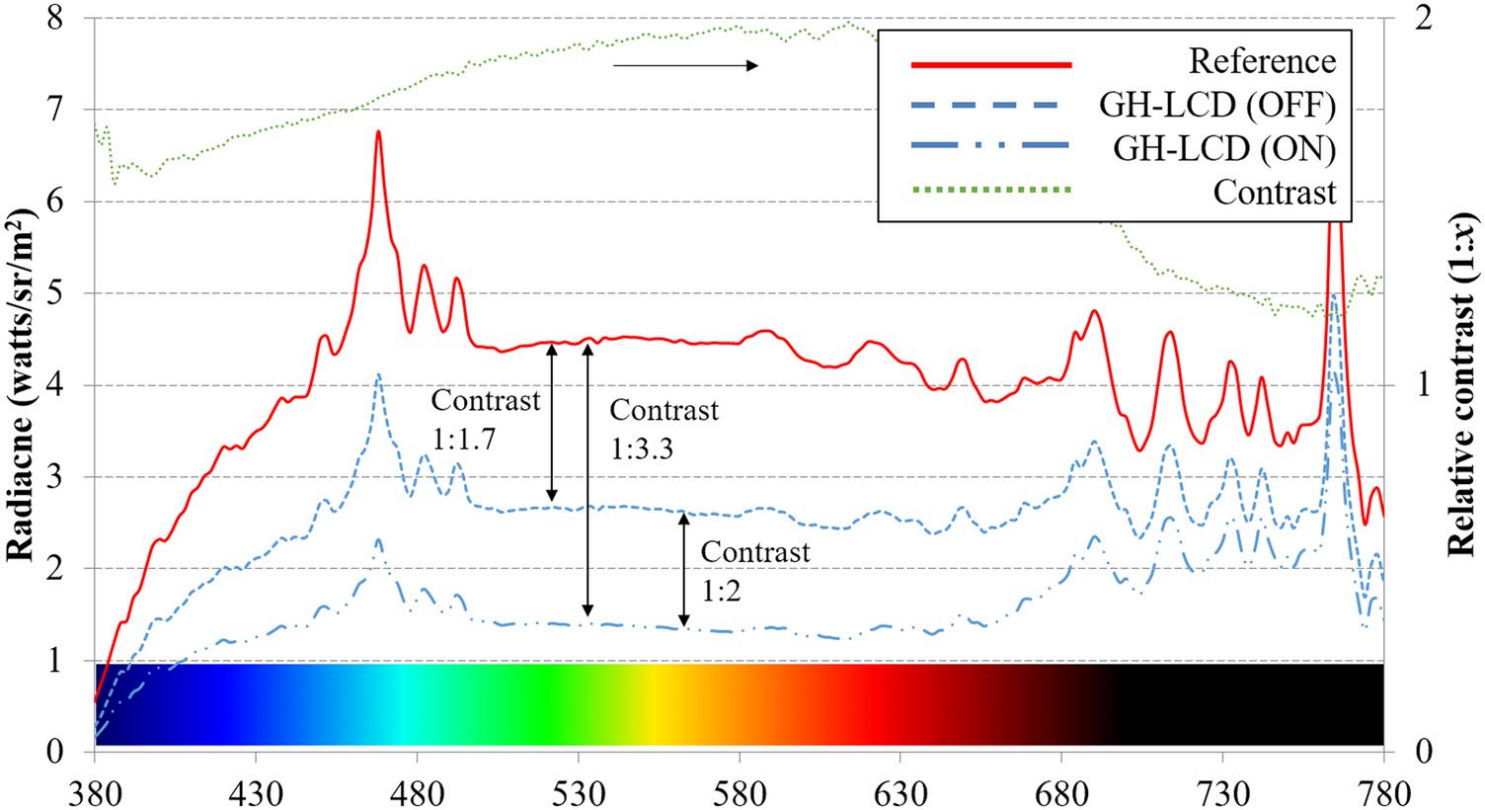
(Left axis) Measured radiance versus wavelength for the reference image (continuous line), active GH-LCD during OFF state (dashed-line) and GH-LCD during ON state (double-dotted line). (Right axis) Calculated contrast using the transmittance values between OFF and ON states of the GH-LCD (dotted-line).

The average radiance values versus light wavelength for the three active cells are shown on the left axis in Fig. 2 (reference, cell OFF and cell ON are represented by a continuous line, dashed-line and double-pointed line, respectively), while the calculated relative contrast is shown on the right axis (dotted-line). It is observed that the active cells have its highest relative contrast around 580 nm with a value of 1:2, and an absolute contrast of 1:3.3. The light attenuation is relatively constant between 480 and 680 nm which corresponds well to the attenuation effect of a neutral black dichroic dye. This analysis confirm a relative contrast of 1:2 with respect to the VLT measurements. For this reason, this value 1:2 was taken for the visual simulations.

### Smart contact lens modelling: customized eye models

Using the OCT-data collected for each eye, customized models were built to match the optimum scleral contact lens design. Scleral fitting characteristics were considered due to the unique stability on-eye during contact lens wear, lack of movement with blinking and downward gaze and the great advantages to incorporate the artificial iris in the optic zone. The foreseen maximum thickness of the artificial iris based on GH-LCD ranges from 100 to 150 μm, while the thickness of the smart insert including the respective electronics and driving components is between 200 and 300 μm, which is compatible with conventional large scleral lenses (with typical thicknesses of 500 μm at the center^33^. The simulated design comprises a total thickness of 520 μm, including a smart insert of 200 μm, and 160 μm of back and front distance to the lens. To minimize the impact of astigmatism and spherical aberration, the design data of the optimum contact lens design of the anterior surface (biconicoid fitting) were: 7.8 mm (radius) and − 0.95 (conic constant) in the horizontal meridian and 7.85 mm (radius) and − 0.8 (conic constant) in the vertical meridian for the right eye (OD); 7.8 mm (radius) and − 0.9 (conic constant) in the horizontal meridian and 7.9 mm (radius) and − 0.85 (conic constant) in the vertical meridian for the left eye (OS). These values correspond to the anterior surface of the contact lens fitted to a biconicoid and used in our patient-specific eye model (Supplementary Table S1). The posterior surface of the contact lens remains with a sphere geometry. The transmission profile of the smart contact lens with artificial iris (65%-pupil center and 32%-pupil periphery) modified the percent efficiency in the retinal plane: 55.2% (6-mm), 47.5% (5-mm), 41.2% (4-mm), 36.3% (3-mm) and 32.7% (2-mm); this filtering could be effective to reduce the light intensity in the eye, critical in aniridia patients. On a healthy eye the pupil diameter changes according to the amount of light the eye is exposed to (i.e. smaller pupil size for brighter light and vice-versa)^34,35^. On average these values are reproducible even among different groups of patients^36^. Table 1 shows the luminance values and corresponding healthy pupil diameters from 6 to 2 mm. Additionally, the calculation of the estimated retinal illuminance (in Troland [cd/m^2 ^ × mm^2^])^37^ for: a healthy eye, an aniridia eye with big pupil and an aniridia eye with a GH-LCD active light filter (transmission profile: 65%-pupil center and 32%-pupil periphery) are presented. On one hand, it is noted that the retinal illuminance of the aniridia eye is higher due to the lacking of iris protection, when compared to the healthy eye. On the other hand, retinal illuminance values of the GH-LCD protected eye are lower for larger diameters (5–6 mm), and higher for diameters below 4 mm. This shows that the current transmission profile allows too much light at high luminance values compared to a healthy iris, which blocks close to 100% of the incoming light.

**Table 1:** Relationship between pupil diameter and luminance, and estimated retinal illuminance for a healthy eye, an aniridia eye (big pupil) and an aniridia eye with a GH-LCD filter.

Diameter (mm)	Luminance (log(cd/m^2^))	Retinal illuminance [Troland]		
		Healthy eye	Aniridia eye	Aniridia eye with GH-LCD
6	− 1.5	0.89	0.89	0.58
5	− 0.6	4.93	7.10	3.90
4	− 0.21	7.75	17.43	8.13
3	0.65	31.57	126.30	50.83
2	5	314,159.26	2,827,433.39	1,008,451.24

### Visual quality and depth of focus for different light level conditions

**Figure 3:**
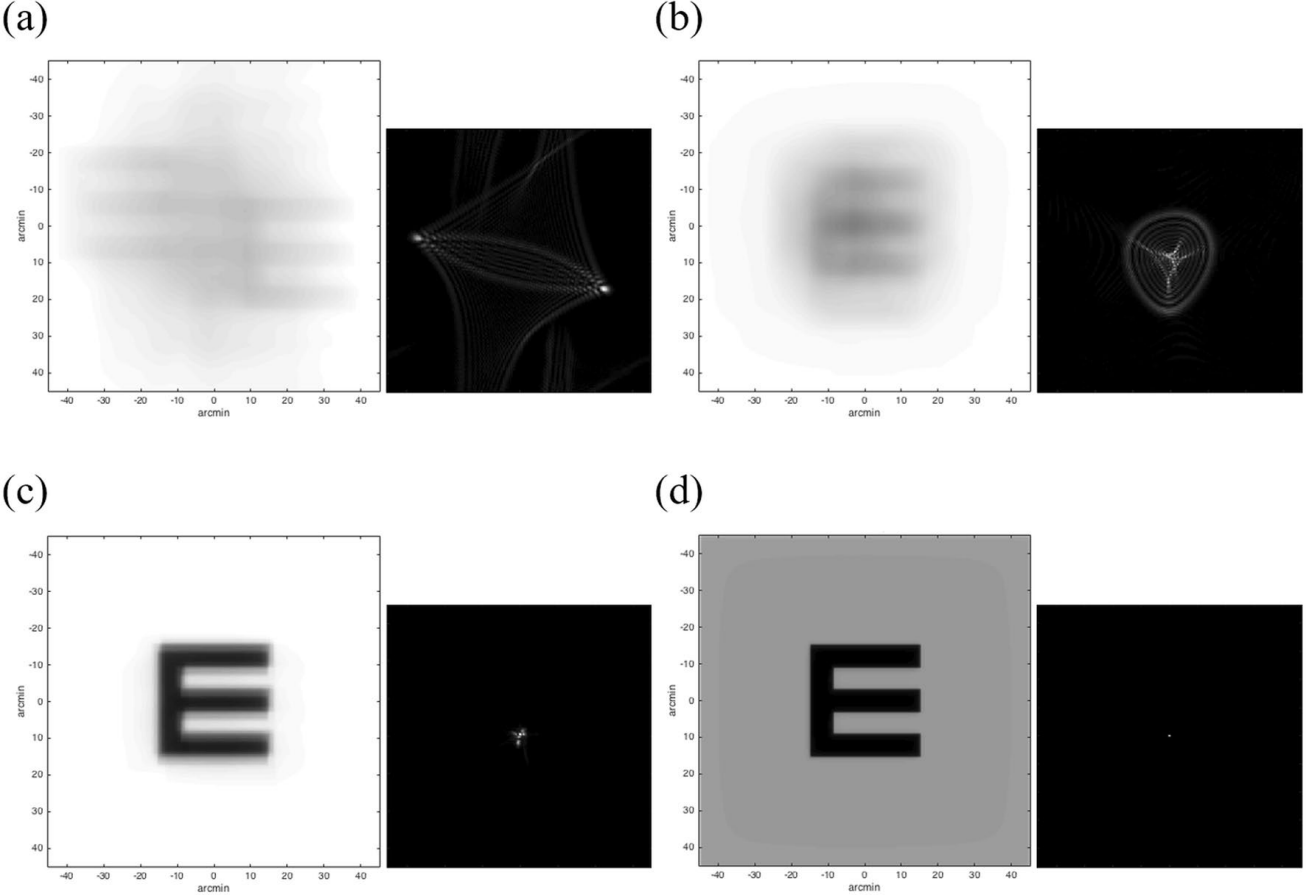
Theoretical simulations of the Snellen E-letter of 30 arc-min and the corresponding PSFs. (**a**) aniridia eye (pupil diameter: 8 mm); (**b**) aniridia eye with a conventional spherical contact lens design (pupil diameter: 8 mm); (**c**) aniridia eye with a customized contact lens design (pupil diameter: 8 mm); (**d**) aniridia eye with the smart contact lens (pupil diameter: 6 mm).

Figure 3 shows the simulated visual acuity of 30-arcmin based on convolution for the aniridia eye (OD) without correction (a) and with different contact lens designs: (b) conventional, (c) customized and (d) customized + artificial iris: smart contact lens). The RMS values of the aniridia eyes without and with the studied contact lens designs (spherical anterior surface, customized anterior surface and customized anterior surface with the artificial iris) are given in Table 2. As expected, the customized smart contact lens design (biconicoid anterior surface) showed a marked decrease in all individual Zernike coefficients; in particular, spherical aberration.

**Table 2:** Root-mean-square (RMS) for high-order aberrations (HOAs), astigmatism, coma, trefoil and spherical aberration.

	EYE (8-mm)	CL sphere (8-mm)	CL best design (8-mm)	Smart CL (6-mm)
**OD**				
HOAs	1.72	1.67	0.28	0.07
ASTIGMATISM	4.67	0.31	0.17	0.09
COMA	0.19	0.13	0.13	0.04
TREFOIL	0.18	0.09	0.09	0.04
SPHERICAL	1.64	1.65	0.16	− 0.01
**OS**				
HOAs	1.91	1.71	0.25	0.08
ASTIGMATISM	4.94	0.46	0.17	0.07
COMA	0.63	0.07	0.07	0.03
TREFOIL	0.42	0.09	0.09	0.06
SPHERICAL	1.70	1.69	0.16	− 0.01

OD, right eye; OS, left eye. EYE, aniridia eye; CL sphere, conventional contact lens design (spherical); CL best design, customized contact lens design considering the best biconicoid fitting; Smart CL, customized contact lens design + artificial iris. Units, microns (μm).

**Figure 4:**
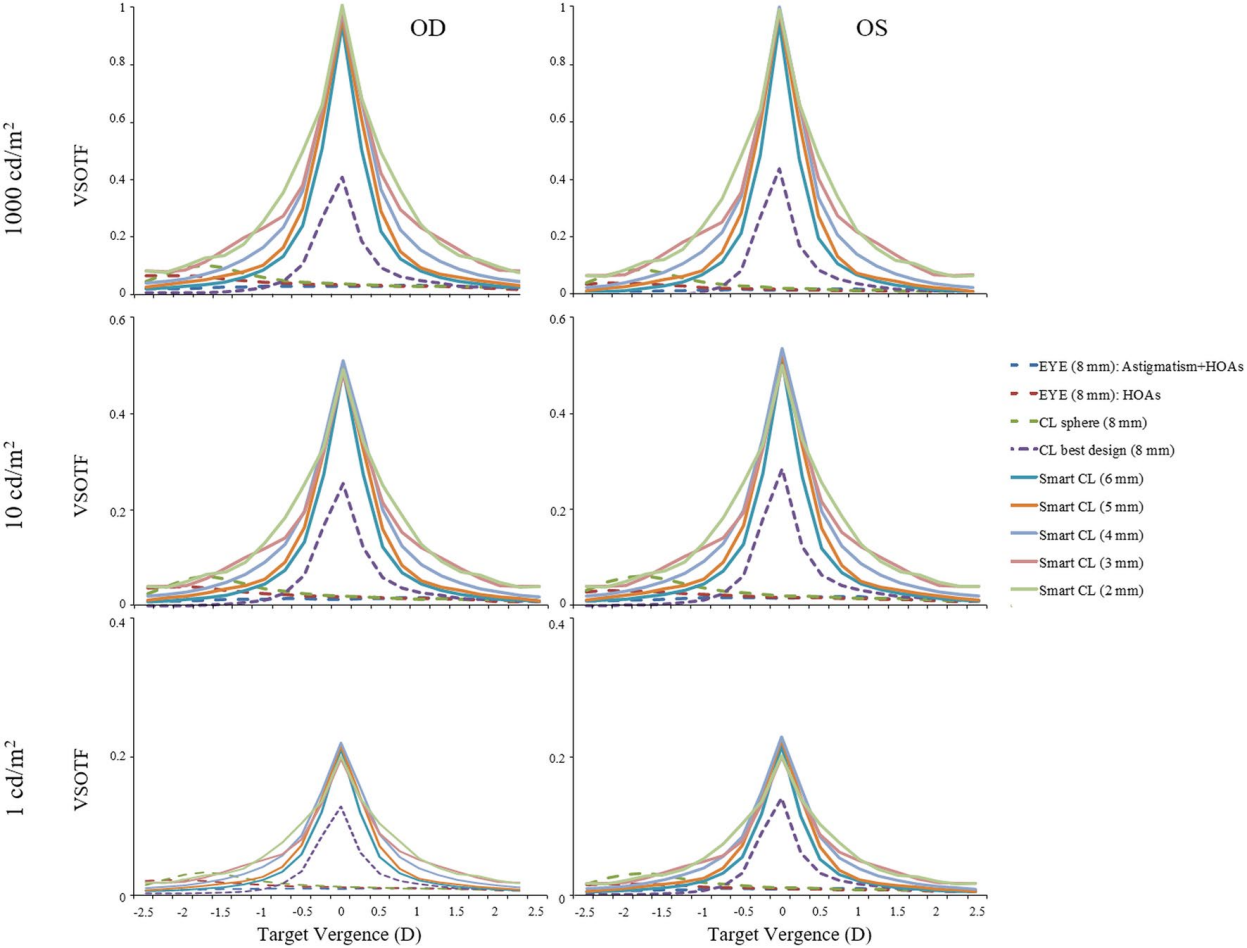
Through-focus plots of Visual Strehl for the aniridia eyes without the smart contact lens (dashed) and with the smart contact lens for a series of pupil diameters (solid) and different light level conditions: (**a**) high photopic—1,000 cd/m^2^; (**b**) low photopic—10 cd/m^2^; and (**c**) mesopic—1 cd/m^2^. HOAs, high-order aberrations; EYE, aniridia eye; CL sphere, conventional contact lens design (spherical); CL best design, customized contact lens design considering the best biconicoid fitting; Smart CL, customized contact lens design + artificial iris.

Figure 4 shows the through-focus Visual Strehl of the aniridia eyes without and with the smart contact lens for different environmental light levels (top, 1,000 cd/m^2^; middle, 10 cd/m^2^; bottom, 1 cd/m^2^). Maximum Visual Strehl was significantly higher with the smart contact lens for high and low light level conditions in all pupil diameters, e.g. OD: 0.86-smart CL 6-mm versus 0.37-CL best design 8-mm (1,000 cd/m^2^), 0.45-smart CL 6-mm versus 0.23-CL best design 8-mm (10 cd/m^2^) and 0.21-smart CL 6-mm versus 0.12 -CL best design 8-mm (1 cd/m^2^). The Visual Strehl shows slight changes with varying pupil diameters × 1.07 (1,000 cd/m^2^), × 1.00-no change (10 cd/m^2^) and × 0.94 (1 cd/m^2^). For the smart contact lens the depth-of-focus yields the maximum value in 2-mm of pupil diameter for both eyes: 3D (1,000 cd/m^2^), 2D (10 cd/m^2^) and 0.75D (1 cd/m^2^)—absolute definition; and 1D for all light levels—relative definition.

## Discussion

This study represents, to our knowledge, the first report of (1) on-bench measurements of light transmission and image quality of an active artificial iris (based on guest–host liquid crystal) and (2) simulations based on real data from an aniridia patient to evaluate the optical performance and depth-of-focus for different light level conditions.

The change in transmittance achieved by guest–host liquid crystal cells (GH-LCD) is here used to directly change the amount of light entering the eye, potentially in an autonomic way (once its combined with the respective driving electronics and power supply). By patterning the cells into concentric rings and actuating them in a consecutive manner, it is possible to mimic the functionality of a healthy human iris. While this actuating configuration has been presented elsewhere, here we focused on the image quality assessment and the impact on an optical system with aniridia characteristics. Although diffraction effects of the spacer distribution was not covered (area coverage of 3.14%), it is estimated that their effect accounts for less than 5% diffraction, when compared to other studies with similar configurations (KAMRA inlay with 5% diffraction and 8.6% of area coverage)^30,32^. The visual simulations and further assessment are based on the measured contrast of the GH-LCD by means of spectral analysis (1:2.1).

Scleral lenses that completely vault the limbus are an excellent option for aniridia patients, since the resting points are beyond the corneal borders. The lens can protect the limbus, provide a therapeutic environment and improve visual quality with a patient-specific design. Furthermore, scleral lenses show unique stability on-eye during lens wear since these lenses are rotationally stable and have lack of movement with blinking and downward gaze^38^. There are different techniques to perform a scleral (mini or large) contact lens fitting. For instance: using scleral topography, corneal topography, optical coherence tomography (OCT) and scleral lens diagnostic fitting sets^39,40^. The most important parameters to consider when fitting a contact lens that rest entirely on the sclera are: the lens total diameter (TD: total diameter at the lens base directly influenced by the horizontal visible iris diameter HVID)^41^, for mini-scleral and large scleral are respectively 15–18 mm and 18.1–24 mm^39^; the central corneal clearance (distance between the cornea and the lens at its center filled with tear fluid), from 200 to 300 μm before the lens settles on the conjunctiva and from 50 to 200 μm afterwards^42^; the lens sagittal height (distance from the lens base to its back curve at the center), which depends on the optical power to correct and geometry of the patient’s eye; and the landing zone design (either symmetric or asymmetric highly dependent on the patient’s eye). Symmetric designs involve spherical or toric designs to properly compensate for astigmatism and toric haptic design for lens rotation and increase the comfort, wearing time and enhance the optical correction^43^. Asymmetric designs deal with regular (quadrant specific scleral lens design) or irregular considerations.

In this publication, we designed and simulated a system with a scleral contact lens thickness and clearance of 520 μm and 250 μm, respectively, which would correspond to an OT_sys_ of 11.1 Barrer.cm^−1^ and pO_2_ of 5.6%. At this level the corneal swelling is only about 3%^44^, while it has been shown that the physiological overnight edema is around 4% (i.e. swelling of a healthy cornea overnight without wearing any type of contact lens)^44^. With this in mind, this low percentage of scleral swelling when wearing the scleral contact lens with the smart platform ensures a safe environment, particularly for short durations. Long term durations are foreseen given that the OT_sys_ is increased above 10% by reducing the clearance and increasing the oxygen permeability of the materials in the stack. It is accepted that the minimum partial pressure of oxygen (pO_2_) at the cornea to avoid any level of edema for more than 6 h is 9.9%^45^.

A link between geometrical factors and optical outcomes may be established by means of a custom selection of the contact lens geometrical parameters. The surface design of the contact lens is paramount in vision correction, and is the compensation of the refractive power of the eye but also have a critical impact in minimizing high-order aberrations (specially, spherical aberration). Hence, surface parameters were virtually calculated by ray tracing to find the curvature of the anterior surface with a biconicoid design (Rx, Qx; Ry, Qy) that minimizes the astigmatism and high-order aberrations. Most high-order aberrations were effectively corrected by a patient-specific scleral lens design and the tear film reservoir between the lens and the cornea, improving by a factor of 6.2 for the same 8-mm pupil (in comparison with a conventional spherical surface). As expected, the high-order aberrations also decreased in the customized design by reducing the pupil size to 6-mm with the artificial iris (× 3.7-RMS of high-order aberrations and × 17-spherical aberration, when compared 8-mm vs. 6-mm). This correction resulted in significant improvement of × 2.2 on average in Visual Strehl [0.41 for 8-mm; 0.89 for 6-mm]. Although scleral lenses are especially suited to minimize corneal astigmatism and high-order aberrations because of their on-eye stability, they tend to decenter. The geometrical characteristics of the sclera (flatter in the nasal side), gravity and eyelids effect usually produces an infero-temporal decentration (on average: 0.91-mm, inferior; 0.62-mm, temporal^46^). The presence of decentration increased astigmatism and high-order aberrations (astigmatism: 0.09 μm vs. 0.19 μm (centered vs. decentered); high-order aberrations: 0.07 μm vs. 0.14 μm (centered vs. decentered), for the smart CL 6-mm OD). To overcome this issue, some scleral lens manufactures induced a decentration in the optic zone.

Altering the real components of the eye’s pupil function—the transmission function (through pupil amplitude apodization)—have been previously studied to optimize the through-focus optical quality and expand the depth-of-focus^47–49^. However, there is an intrinsic reduction in retinal illuminance with pupil apodization due to an attenuation of high spatial frequencies^49^. In addition, the reduced retinal illuminance associated with small pupils elevates contrast thresholds associated with photon noise. The active GH-LCD cells reduce uniformly the mean luminance of the scene, producing a mean transmittance attenuation factor of 1:2.1 when looking through the active cells. The transmission profile of the simulated smart contact lens with the artificial iris showed different magnitudes of intensity transmission profile for different pupil sizes (55.2%—6 mm; 32.7%—2 mm). Retinal illuminance values of the GH-LCD protected eye compared to a healthy eye (which blocks to 100% of the incoming light) are lower for larger diameters (5–6 mm), and higher for diameters below 4 mm. This shows that the current transmission profile allows too much light at high luminance values. Higher contrast ratios of the GH-LCD would improve this situation and provide higher protection against light. The consequent loss in contrast ratio caused a decrease in retinal image contrast (affecting both the background and the stimulus as we can observe in Figs. 1 and 3); however, this degradation was minimized by a patient-specific scleral lens design upon compensation of high-order aberrations. An automatic actuation approach, in which the retinal illuminance is controlled based on the experienced luminance is favorable for the patient, compared to a manually activated device. Automatic actuation by means of a photovoltaic prototype cell and a custom-made TFT LC driver chip has been presented as potential solution^8^.

We also introduced into the model the impact of the artificial pupil size and environmental light level conditions to evaluate the optical quality (in terms of Visual Strehl) and the theoretical near vision of the smart contact lens in these eyes. Our estimates of depth-of-focus show functional near vision under photopic illumination for pupils < 3.0 mm, with 3D of depth of focus for 3-mm and 2-mm pupil diameters under illumination of 1,000 cd/m^2^, and 2D and 1.5D for 3-mm and 2-mm pupil diameters, respectively, under low photopic light levels (10 cd/m^2^). Our results represent a clinically relevant increase in depth-of-focus and hold similarities with those recently reported about the interaction of aberrations and quantal fluctuations in visual quality for different pupil sizes^50^, opening therefore a great variety of possibilities for presbyopic patients. In daily life, individuals operate in a visual environment that varies in luminance across the day by as much as 100,000:1; therefore, future studies on patients are essential to confirm the experimental and theoretical results and shed light about the dynamic light attenuation and visual performance of the active GH-LCD, especially in low-photopic and mesopic light levels.

Our study did not directly address a comprehensive visualization of crystalline lens opacities in the aniridia patient; however, OCT also allowed the three-dimensional imaging of the crystalline lens. Transparency of the crystalline lens is due to the specific organized distribution of the fibers and high concentrations of crystalline proteins in the lens fibers, which limits light-scattering and absorption. In eyes with cataract, scattering by the opacified lens causes a major decrease in image quality and it is associated with age; however, although is not the main cause of low vision, people with aniridia usually develop cataract at an early age. In this patient, we can specifically observe the peripheral regions of the crystalline lens (limited by the iris in normal subjects) and how both eyes showed the same morphological opacification pattern of the posterior crystalline lens. In aniridia, the retinal irradiance cannot be controlled, so the excessive amounts of light entering the eye might be associated with cataract progression in adolescence. Consequently, the light transmission configuration of the artificial iris (65%-pupil center and 32%-pupil periphery) will control automatically the light propagation and retinal illuminance under the smart contact lens correction and could have a benefit by attenuating the light intensity through the ocular media, achieving secure levels of retinal illuminance and reducing glare.

To sum up, we have presented a smart contact lens with adjustable light transmission to modulate the incoming light as an artificial iris and a potential solution to presbyopia by expanding the depth-of-focus to 3D under photopic light levels, achieving a vision correction that will surpass current optical solutions. This technology can potentially be validated throughout clinical investigations with patients/volunteers under clinical conditions (hospital environments). After the biocompatibility and safety test have been cleared out, the clinical protocol would consider the evolution of visual acuity, contrast sensitivity and light sensitivity before and after wearing the investigational device (i.e. GH-LCD based contact lens). In practice, these tests can be split in two phases: passive (where a constant light filter would mimic the GH-LCD) and active (where the GH-LCD in combination with the respective electronics are included).

## Methods

### Active GH-LCD cells

**Figure 5:**
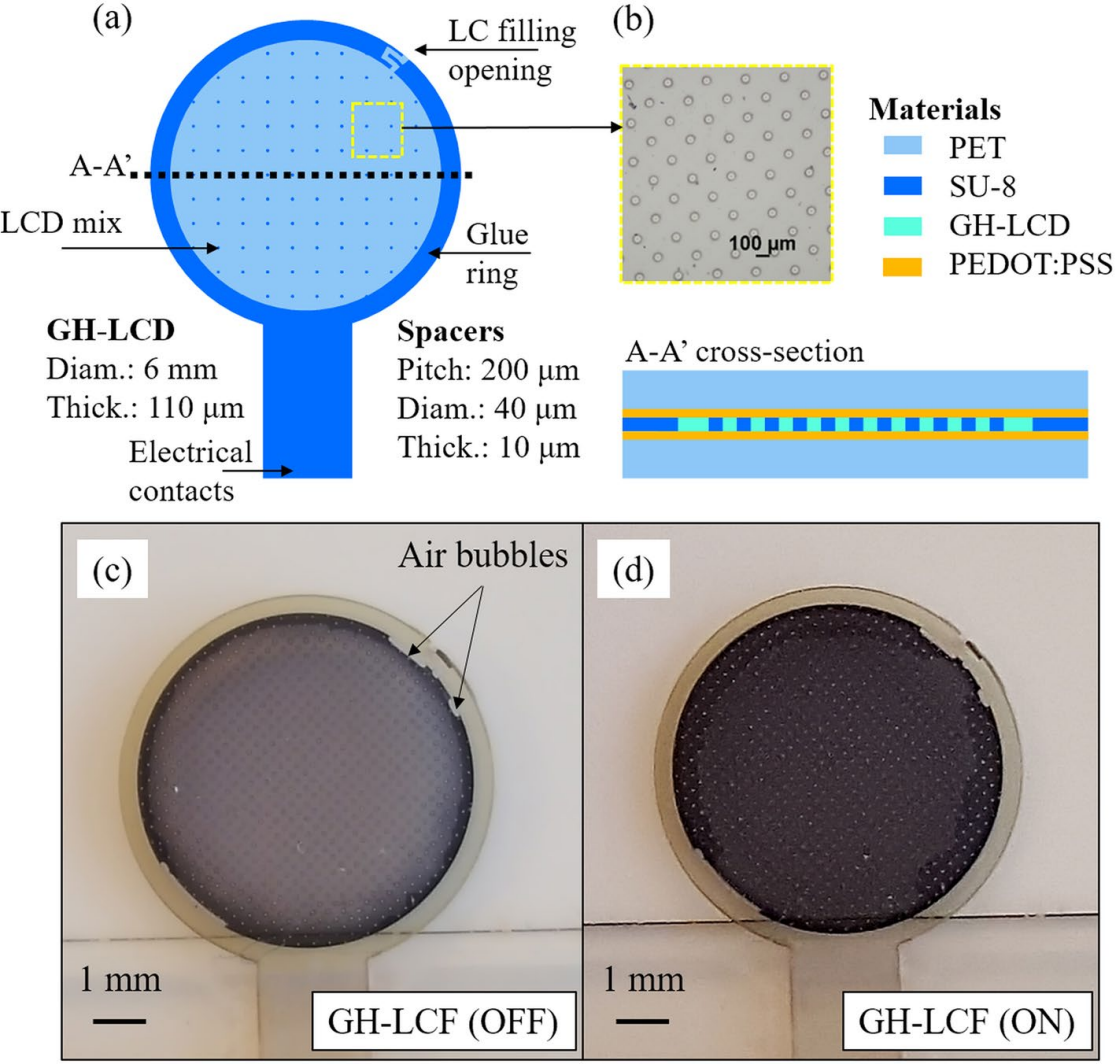
(**a**) Layout of the GH-LCD cells including spacers, ring of glue and liquid crystal filling opening; (**b**) optical microscope images of the spacers and cross-section detail of the GH-LCD cells; (**c**,**d**) optical microscopy images (magnification 5×) of the GH-LCD cells after filling without and with electrical driving (1 kHz @ 20V_pp_), respectively.

The GH-LCD was fabricated on polyethylene terephthalate (PET) substrates of 50 μm-thick, separated by 10 μm-thick SU-8 series 2000 (from MicroChem) cylindrical spacers realized by a photolithography process^51^. The distribution of the pillars is important in order to achieve a homogeneous gap between the top and bottom electrodes, especially when using thin substrates (< 100 μm). For this work, a pitch of 200 μm with a square distribution pattern was used. The pillars have a diameter of 40 μm with a thickness of 10 μm. This pattern accounts for an area coverage of ${(20 \upmu \mathrm{m})}^{2}\pi /{(200 \upmu \mathrm{m})}^{2}=3.14\%.$ Transparent PEDOT:PSS poly(3,4-ethylenedioxythiophene) polystyrene sulfonate (from Sigma Aldrich) was spin-coated with a thickness of 100 nm onto the cleaned PET substrates to serve as conductive electrodes. The GH-LCD mix was composed of the liquid crystal MLC-6608 (from Merck), the chiral dopant S811 (from Merck) and a neutral black dichroic dye (3 wt%). An alignment layer of 6 nm-thick SiO_2_ was evaporated onto the conductive layers and pre-defined spacers to determine an initial orientation of the liquid crystal at the surfaces. The cells were filled by means of custom-made vacuum filling process, described elsewhere^24^. The glue UVS 91 (from Norland Products Inc.) was used to close the liquid crystal filling opening. Figure 5a,b show the layout design and optical microscope images of the GH-LCD cells (diameter: 6 mm). This active area is intended to be combined with a fixed light filter extending from 6 mm to at least 10 mm, in order to cover the whole range of the pupil and offer higher light protection. Beyond 6 mm the effect of a dynamic artificial iris is much smaller since this corresponds to relatively dark environments (i.e. lower than 0.01 lx). Figure 5c,d show the images of the cell when OFF and ON, respectively. The cells are electrically driven by a square wave with an amplitude of 20 Vpp, frequency of 1,000 Hz and zero bias voltage. A periodic pulsed driving voltage is desired in order to prevent electrolysis due to direct voltage which would cause the electrodes to become coated and destroy the display^4,8,25^. The frequency should be higher than 60–100 Hz to prevent flickering but lower enough to prevent the reduction of input impedance and high power consumption. Additionally, a squared signal symmetrical with respect to ground (50% duty cycle) was used in order to avoid any damaging DC offset voltage.

Considering the highly restrictive space within an implementation of a smart contact lens^13^, the GH-LCD driver needs to comply with low rigidity, small footprint, custom dimensions and low power consumption. As example, this was achieved when using the amorphous indium gallium zinc oxide (a-IGZO) self-aligned thin-film transistor (TFT) on polyimide technology, as presented in^8^. In that particular implementation, the required voltage level for a GH-LCD component at 1 kHz was achieved by means of an H-bridge with a power consumption and area under 25 μW and 1 mm^2^, respectively. A proof-of-concept photovoltaic cell was used in combination with the GH-LCD driver to illustrate possible sources of power for the smart contact lens platform^8^. Further optimizations with respect to power consumption and available channels (i.e. for more LDC segments) are needed in order to realize a fully autonomous device.

Following its fabrication, the smart platform (GH-LCD and electronics^8,13^) would be embedded inside a conventional rigid gas permeable scleral contact lens to protect the eye from the device and vice-versa. This process could be achieved by a custom-made double lathe diamond tool process, where first each half of the lens is lathed with a cavity to fit the platform, then the three parts are mechanically fixed (i.e. biocompatible glue mix), and finally, the base and front curves of the lens are machined. Especial attention needs to be placed to the roughness and continuity of the base lens in contact with the eye.

The amount of oxygen reaching the cornea is crucial to prevent corneal edema (swelling of the cornea due to hypoxia)^44^. The equivalent oxygen percentage (EOP) method can be used to estimate the partial pressure in oxygen (pO_2_) at the corneal surface. The latter depends on the properties of the contact lens material (i.e. thickness and oxygen permeability) and clearance (i.e. distance from the lens to the cornea filled with tear fluid). This technique expresses relative corneal surface pO_2_ in between the lens and cornea, and compares it to the relative pO_2_ in the atmosphere (20.9%)^52^. Equation (1) presents the nonlinear regression obtained from clinical data relating the pO_2_ to oxygen transmissibility (Dk/t)^52,53^.

1 $$pO_{2} = - 19.6 \times exp^{{\left( { - 0.029 \times Dk/t} \right)}} + 19.8 \left[ \% \right]$$

The oxygen transmissibility of the complete system (OT_sys_) including the contact lens and tear fluid, commonly known as Dk/t and measured in Barrer/cm, can be calculated using a diffusion law similar to Ohm’s law for series resistances (i.e. total resistance to the diffusion of oxygen through the system). The OT_sys_ can be then defined as the reciprocal of that resistance^54^. Equation (2) presents the OT_sys_ calculation considering Dk_1_/t_1_ the scleral lens and Dk_2_/t_2_ the tear layer trapped between the cornea and the lens^55^. Dk_1–2_ and t_1–2_ are the oxygen permeability and central thicknesses of the scleral lens (125 Barrer) and tear fluid (80 Barrer), respectively.

2 $$OT_{sys} = \left( {Dk/t} \right)_{sys} = \frac{1}{{\left( {t_{1} /Dk_{1} } \right) + \left( {t_{2} /Dk_{2} } \right)}} \left[ {\frac{{{\text{Barrer}}}}{{{\text{cm}}}}} \right]$$

**Figure 6:**
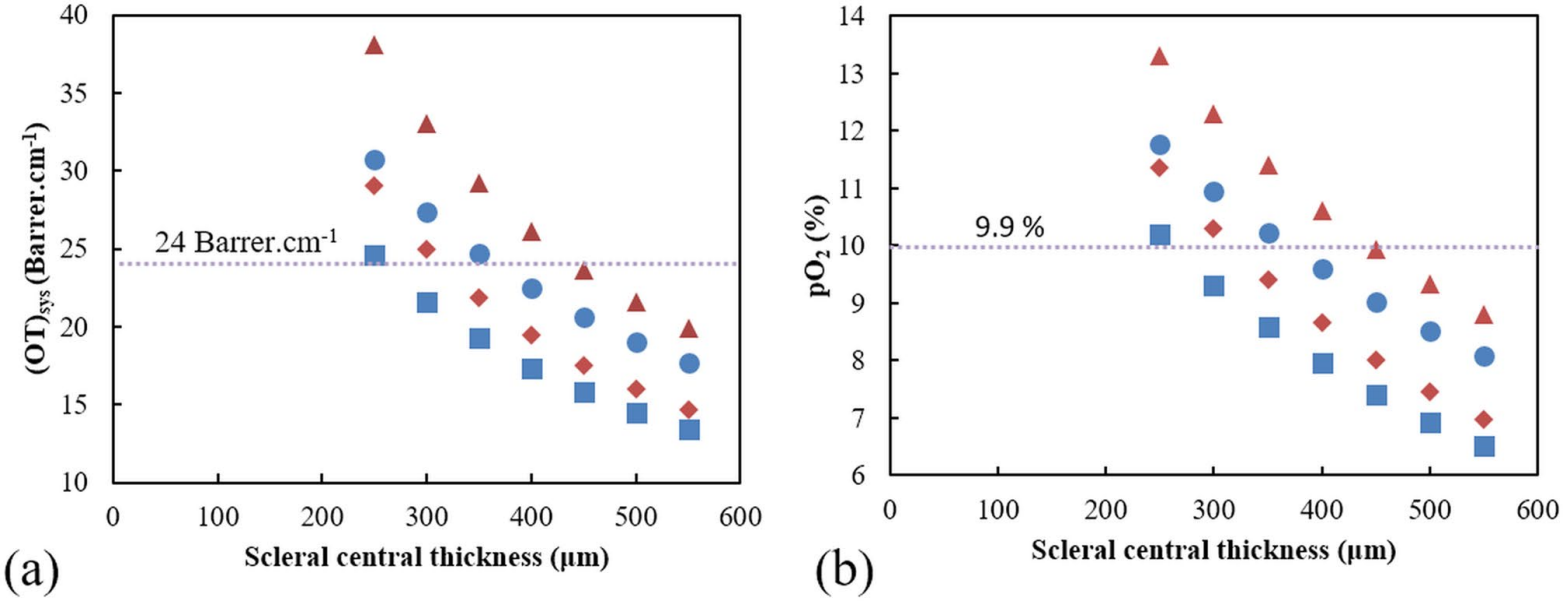
(**a**) Oxygen transmissibility of the system (scleral contact lens + tear fluid) in [Barrer/cm] for different scleral contact lens central thicknesses (from 250 to 550 μm); (**b**) partial pressure in oxygen in between the scleral contact lens and cornea. Diamond and triangle represent the values for a tear fluid of 50 μm-thick, with and without the smart insert, respectively; while square and circle represent the values for a tear fluid of 100 μm-thick, with and without the smart insert, respectively.

The GH-LCD presented here is compatible and can be integrated with the smart platform presented in^13^, in order to provide appropriate energy through RF wireless protocols. The OT of the complete system including: tear fluid Dk_2_/t_2_ and smart platform (i.e. GH-LCD, thermoplastic polyurethane—TPU and electronics interconnections) inside the scleral contact lens Dk_1_/t_1_ was estimated by using Eq. (2). The latter one was calculated as an effective value considering the coverage area and oxygen permeability of each component, thus each material. The scleral lens material: 125 Barrer with a 55% coverage area, and the TPU material: 80 Barrer^56^ with a 25% coverage area. The other components such as the GH-LCD were assumed to have 0 Barrer thus no oxygen permeation. Besides material properties, oxygen transmission can be increased by means of perforations to the smart platform. These perforations can take place on the surroundings of the GH-LCD^13^ or directly on the GH-LCD cell^25^. On one hand, the first approach will provide a direct contact with the RGP material (with an oxygen permeability—Dk higher than 125 Barrer) and enable the mechanical stability required for a thermoforming process. On the other hand, small perforations (e.g. 10 μm in diameter) located at the separation pillars by means of laser ablation would allow oxygen to go through the otherwise impenetrable GH-LCD cell. However, the small perforations would impact the optical quality of the lens by introducing haze and diffraction. Figure 6a shows the OT_sys_ for two clearance values (i.e. 100 μm and 50 μm) and different scleral lens central thicknesses (i.e. ranging from 250 to 500 μm), with (diamond) and without (triangle) the smart insert. Figure 6b shows the pO_2_ for the same thicknesses values, with (diamond) and without (triangle) the smart insert, respectively.

### Experimental validation

The quantitative optical quality of the active GH-LCD cells (ON/OFF states) and visible light transmittance (VLT) was experimentally obtained by means of a white-light based optical setup. Three GH-LCD were measured for statistical purposes and a baseline measurement (without GH-LCD cells). The system comprises an illumination channel (Hamamatsu Super-Quite Xenon Lamp, model L2175 150 W; housing model E7536, power source model C8849), which emits a stable power in the spectral range between 185 and 2000 nm, optical elements required to collimate the beam and generate an uniform irradiance across the samples including two converging lenses (f′ = 75 mm) and a pinhole diaphragm with a diameter of 8 mm. A specific stimulus (glass/chromium mask with fully opaque features of 200 μm) was placed in the conjugated plane and its image was directly projected on a CMOS color camera (DCC1645C from Thorlabs) for further analysis of their average transmittances. The raw data was saved as Tagged Image File Format images while the color composition (RGB from 0 to 255) was measured along a horizontal axis with the histogram function of the ThorCam software application to calculate the respective contrast values. These values were used as input parameters of the smart contact lens optical model in order to evaluate the theoretical visual performance.

### Spectral measurements

The spectrum analyzer SpectraScanPR-670 (from PHOTO RESEARCH) in combination with the previously described white-light based optical setup was used to characterize the spectral profile of the active GH-LCD cells (ON/OFF states). The mask stimulus was removed and the CCD camera was replaced by a Lambertian surface at the end of system. The analyzer was placed at a distance of 2 m and its objective was directed to the dispersion substrate with an aperture fixed at 0.25°, while the experiment room was completely dark. The GH-LCD contrast 1:*x*, where *x* is the ratio between OFF transmittance and ON transmittance, was calculated based on the measured radiance [watts/sr/m^2^] between 380 and 780 nm. Finally, the comparison to the baseline measurement allowed to point out differences between the light filtering performances of the GH-LCD cell states (ON and OFF).

### Three-dimensional measurements of the anterior segment in an aniridia patient: Optical Coherence Tomography Imaging

**Figure 7:**
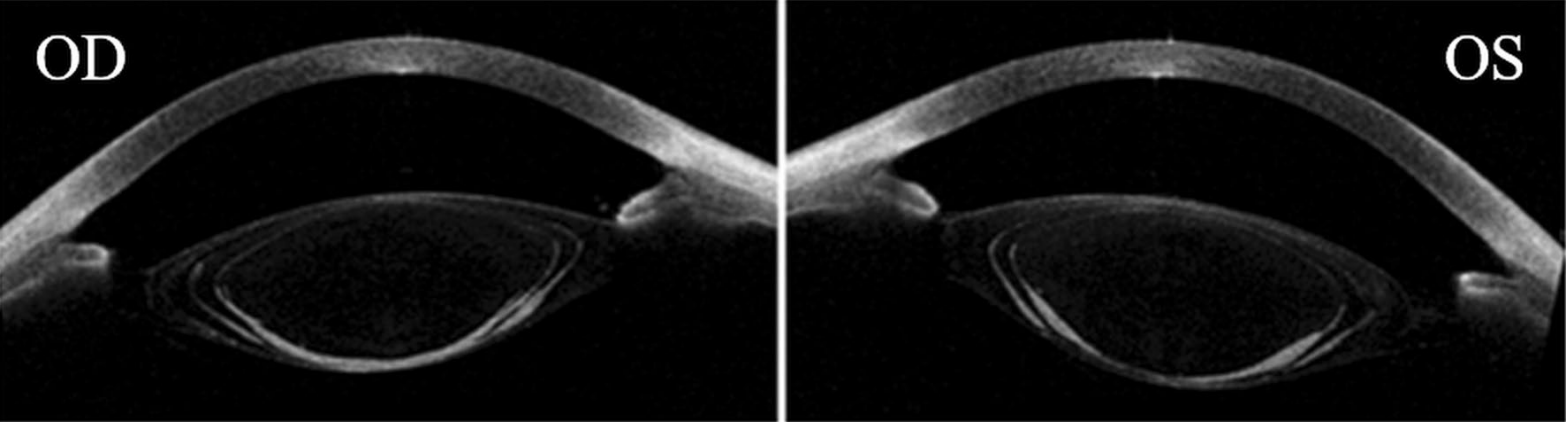
Cross-sectional OCT image of the aniridia patient (OD, right eye; OS: left eye).

The Casia 2 (from Tomey Corp., Nagoya, Japan) uses 1,310 nm high-speed swept-source OCT technology. Images were generated using a rate of 50,000 A-scans per second with a 16 mm diameter scanning range. For assessment of the anterior segment, 16 radial cross-sectional images were obtained in 0.3 s. Figure 7 shows one cross-sectional image of both eyes. The OCT measurements were performed in an 18-year-old female aniridia patient.

### Three-dimensional surface data analysis and optical quality metrics: smart contact lens simulations on an aniridia patient

OCT-based elevation data from both anterior and posterior corneal surfaces of the aniridia patient were fit to Zernike polynomial expansions (note that these are fits to surface elevations not corneal wave aberrations) using custom routines written in Matlab (MathWorks, Natick, MA). The pupil center was used to define the center of the optical zone, and used as a reference. The Zernike coefficients of the anterior and posterior corneal surfaces (see Supplementary Table S1), anterior segment distances and crystalline lens geometry were exported to ZEMAX (Focus Software, Tucson, Arizona, USA) for virtual ray tracing analysis^57^.

Matlab was used to create a suitable input file into ZEMAX (ZEMAX DDE toolbox). The corneal refractive index was taken as 1.376, the aqueous humor as 1.336 and the crystalline lens refractive index was taken as a constant (n = 1.422). For the contact lens we used (1) the nominal values of a conventional scleral contact lens (CL sphere [Misa lens from Microlens BV Netherlands]): 9.46 mm (radius of the anterior lens surface), 9.3 mm (radius of the posterior lens surface) and 0.3 mm (thickness); and (2) the customized smart contact lens with the artificial iris (CL best design): variable anterior surface data (considering the best biconicoid fitting in 0.05 mm-step (radius) and 0.05-step (conic constant)—to compensate astigmatism and spherical aberration of the aniridia eye-), 9.3 mm (radius of the posterior lens surface), 9.3 mm (radii of the anterior and posterior surfaces of the artificial iris), 0.52 mm (smart contact lens thickness: 0.16 mm, anterior lens-anterior iris; 0.2 mm, artificial iris; 0.16 mm, posterior iris-posterior lens). The refractive indices were: 1.43-contact lens, 1.43-artificial iris, 1.336-tear film; and light transmission: 65%-pupil center and 32%-pupil periphery. The last two were found experimentally as described in the section: “Qualitative optical quality and visible light transmittance (VLT)”. The transmittance of the GH-LCD is limited (even in the OFF state) by the transmittance values of the materials present in the stack: PET substrate, PEDOT:PSS conductive layer, SiO_2_ alignment layers and absorption of the dichroic molecules mixed in the liquid crystal. It has been shown that the transmittance of PET substrates (250 μm) is above 90%^58,59^, while the value for a 100 μm-thick PEDOT:PSS film is around 87.7%^60^. The absorption of the dichroic dye when aligned vertically is considered as a small percentage and the effect of 6 nm of SiO_2_ was neglected. Considering those values the VLT of the stack is approximately 64% (i.e. 2 × 92% from PET, 2 × 87.7% from PEDOT:PSS and 2% from dye), in accordance with the experimental values.

Wave aberrations were calculated for monochromatic light (555 nm), by tracing an array of 64 × 64 rays within the central pupil diameter area (from 8 to 2 mm-pupil diameter) through a 4-surface eye model for the aniridia patient and with a 8-surface model for the aniridia patient with the smart contact lens. Wave aberrations were described in terms of individual Zernike coefficients.

Optical quality was described in terms of Visual Strehl, as it has been shown to hold the highest correlation variance against subjective visual acuity^61^. Visual Strehl was computed as the volume under the Visual Optical Transfer Function (obtained from the overlapping of the Optical Transfer Function (OTF) with the inverse of a general Neural Transfer Function for different environmental light levels: 1,000, 10 and 1 cd/m^2^)^50^, normalized to diffraction limit. Visual Strehl was evaluated through focus, in 0.25 D defocus steps. The maximum value of the through-focus Visual Strehl curve was obtained as the best corrected optical quality metric. Depth of focus was defined as the dioptric range for which Visual Strehl was at least 50% the maximum Visual Strehl value in the through-focus curve (relative definition) and as the dioptric range for which Visual Strehl was above 0.12 (absolute definition). To assess the interaction of transmission function of the artificial-iris´s pupil function, the Visual Strehl was calculated with various magnitudes of intensity transmission profiles for different pupil areas.

## Supplementary information

Supplementary Information.

## Data Availability

All data generated or analyzed during this study are included in this published article.
