# Supplementary material for: Artificial iris performance for smart contact lens vision correction applications
Source: Sci Rep. 2020 Sep 4;10:14641. doi: 10.1038/s41598-020-71376-1 (PMC7474060; doi:10.1038/s41598-020-71376-1)
Supplement: Supplementary file 1 — Supplementary Information. [file 41598_2020_71376_MOESM1_ESM.docx]

Artificial iris performance for smart contact lens vision correction applications

# Andrés Vásquez Quintero*^1^, Pablo Pérez-Merino^2^, Herbert De Smet^1^

# 1 Ghent University and imec, Centre for Microsystems Technology (CMST), Technologiepark 126, 9052 Ghent, Belgium

# 2 Instituto de Investigación Sanitaria Fundación Jiménez Díaz,

# Avda. Reyes Católicos 2, 28040 Madrid, Spain

*Corresponding author: Andrés Vásquez Quintero

Contact information:

Ghent University and imec, Technologiepark 126, 9052 Ghent, Belgium

Email: [andres.vasquez@ugent.be](mailto:andres.vasquez@ugent.be), Tel: +32 (0) 9264 66 24

**Supplementary information**

**Supplementary Table S1.** Second- through sixth-order Zernike coefficients (ANSI Standard Convention) of the anterior and posterior corneal surfaces for an 8-mm pupil diameter. OD: right eye; OS: left eye.

|  | **OD** | | **OS** | |
| --- | --- | --- | --- | --- |
|  | *Anterior* | *Posterior* | *Anterior* | *Posterior* |
| **Z2,-2** | -0.00806 | -0.01402 | 0.00651 | 0.01256 |
| **Z2,0** | 0.32186 | 0.40677 | 0.31998 | 0.40561 |
| **Z2,2** | -0.01318 | -0.02297 | -0.01515 | -0.02905 |
| **Z3,-3** | -0.00030 | 0.00233 | -0.00108 | -0.00160 |
| **Z3,-1** | -0.00154 | -0.00629 | -0.00237 | -0.00523 |
| **Z3,1** | 0.00046 | 0.00162 | 0.00142 | 0.00273 |
| **Z3,3** | 0.00005 | 0.00065 | 0.00029 | -0.00199 |
| **Z4,-4** | -0.00031 | 0.00006 | 0.00041 | 0.00037 |
| **Z4,-2** | 0.00002 | -0.00024 | 0.00039 | 0.00103 |
| **Z4,0** | 0.00429 | 0.00798 | 0.00445 | 0.00803 |
| **Z4,2** | 0.00052 | 0.00179 | -0.00009 | 0.00132 |
| **Z4,4** | 0.00033 | -0.00056 | -0.00017 | -0.00080 |
| **Z5,-5** | -0.00026 | -0.00062 | 0.00014 | 0.00179 |
| **Z5,-3** | -0.00014 | -0.00017 | -0.00035 | 0.00029 |
| **Z5,-1** | -0.00014 | -0.00072 | 0.00002 | -0.00051 |
| **Z5,1** | -0.00050 | 0.00010 | 0.00023 | -0.00010 |
| **Z5,3** | 0.00021 | 0.00048 | -0.00020 | 0.00047 |
| **Z5,5** | -0.00076 | -0.00321 | 0.00022 | -0.00134 |
| **Z6,-6** | -0.00019 | -0.00008 | -0.00056 | -0.00152 |
| **Z6,-4** | 0.00013 | -0.00006 | 0.00015 | 0.00045 |
| **Z6,-2** | 0.00014 | -0.00068 | 0.00014 | 0.00023 |
| **Z6,0** | -0.00046 | 0.00007 | -0.00041 | 0.00000 |
| **Z6,2** | 0.00025 | -0.00075 | 0.00033 | -0.00020 |
| **Z6,4** | -0.00013 | -0.00125 | -0.00021 | -0.00110 |
| **Z6,6** | -0.00044 | 0.00128 | -0.00003 | 0.00125 |
